# Recruited fibroblasts reconstitute the peri-islet membrane: a longitudinal imaging study of human islet grafting and revascularisation

**DOI:** 10.1007/s00125-019-05018-1

**Published:** 2019-11-07

**Authors:** Julia Nilsson, Rabiah Fardoos, Lisbeth Hansen, Håkan Lövkvist, Kristian Pietras, Dan Holmberg, Anja Schmidt-Christensen

**Affiliations:** 1 Department of Experimental Medical Science, Lund University, 221 84 Lund, Sweden; 2 Lund University Diabetes Centre, Malmö, Sweden; 3 Department of Clinical Sciences, Lund, Neurology, Lund University, Lund, Sweden; 4 Clinical Studies Sweden – Forum South, Unit for Medical Statistics and Epidemiology, Skåne University Hospital, Lund, Sweden; 5 Division of Translational Cancer Research, Department of Laboratory Medicine, BioCARE, Lund University, Lund, Sweden

**Keywords:** Anterior eye chamber, Diabetes mellitus, Extracellular matrix, Fibroblast, Human islet, Islet transplantation, Longitudinal imaging, Peri-islet membrane, Post-transplantation period, Vascularisation

## Abstract

**Aims/hypothesis:**

Rapid and adequate islet revascularisation and restoration of the islet–extracellular matrix (ECM) interaction are significant factors influencing islet survival and function of the transplanted islets in individuals with type 1 diabetes. Because the ECM encapsulating the islets is degraded during islet isolation, understanding the process of revascularisation and engraftment after transplantation is essential and needs further investigation.

**Methods:**

Here we apply a longitudinal and high-resolution imaging approach to investigate the dynamics of the pancreatic islet engraftment process up to 11 months after transplantation. Human and mouse islet grafts were inserted into the anterior chamber of the mouse eye, using a NOD.*ROSA-*tomato.*Rag2*^−/−^ or B6.*ROSA-*tomato host allowing the investigation of the expansion of host vs donor cells and the contribution of host cells to aspects such as promoting the encapsulation and vascularisation of the graft.

**Results:**

A fibroblast-like stromal cell population of host origin rapidly migrates to ensheath the transplanted islet and aid in the formation of a basement membrane-like structure. Moreover, we show that the vessel network, while reconstituted by host endothelial cells, still retains the overall architecture of the donor islets.

**Conclusions/interpretation:**

In this transplantation situation the fibroblast-like stromal cells appear to take over as main producers of ECM or act as a scaffold for other ECM-producing cells to reconstitute a peri-islet-like basement membrane. This may have implications for our understanding of long-term graft rejection and for the design of novel strategies to interfere with this process.

**Electronic supplementary material:**

The online version of this article (10.1007/s00125-019-05018-1) contains peer-reviewed but unedited supplementary material, which is available to authorised users.

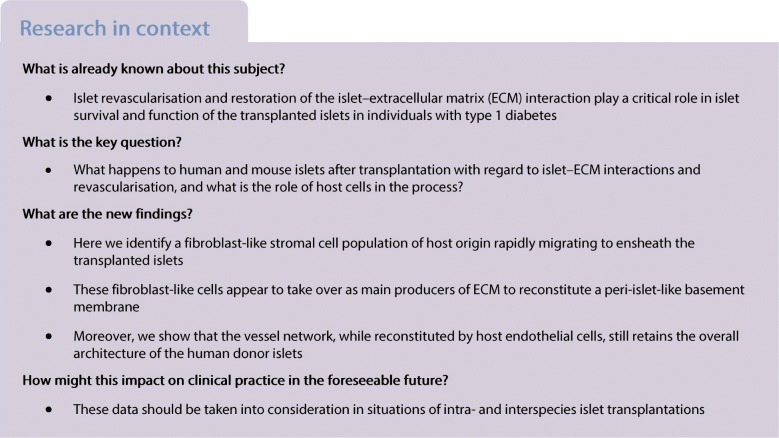



## Introduction

Type 1 diabetes is the result of the autoimmune destruction of insulin-producing beta cells in the pancreas, and usually presents during childhood or young adulthood. Standard treatment of these patients involves exogenous insulin administration, by either (multiple) daily injections or infusions. However, sporadic administration of exogenous insulin often fails to maintain tight glycaemic control, provoking hyperglycaemic and hypoglycaemic episodes that can cause devastating side effects [1, 2]. Beta cell replacement offers the potential to provide physiological glycaemic control, but despite the early promise, islet transplantation as a therapeutic option for type 1 diabetes has had limited clinical impact.

Many factors contribute toward graft failure, including the inflammatory and immunogenic host environment and the loss of cells as a result of ischaemia and inadequate revascularisation [3, 4]. Transplanted islets must adapt to their new surroundings without the internal vascularisation and innervation that they had in the pancreas, and they do not have the benefit of most of their native peripheral extracellular matrix (ECM). An optimal engraftment site requires access to adequate oxygen and nutrient supplies either from endogenous vasculature or from induced or intrinsic neovascularisation [5]. Thus, graft revascularisation plays a critical role in islet viability and function [6], as well as in restoration of the islet–ECM interactions [7–9].

The ECM consists of glycoproteins including fibrillar collagens, proteoglycans and other glycoproteins such as laminins and fibronectin formed into a supportive network that generally acts to separate tissue compartments, while providing specific molecular signals that control processes such as cell migration, differentiation and survival [10–13]. The ECM is present in two forms: basement membrane and interstitial or stromal ECM. Basement membranes predominate in the pancreatic ECM, supporting epithelial acini of the exocrine pancreas and surrounding blood vessels and ensheathing each pancreatic islet (reviewed in [14]).

The pancreatic tissue-specific microenvironment formed by the ECM is partly lost during the islet isolation process [15–17]. The local disruption of the ECM and thereby the integrin-mediated adhesion of the ECM to adjacent islet cells results in apoptosis [18]. Survival is promoted by allowing cells to adhere [19, 20], culturing islets on or within solid ECM protein coated scaffolds [10, 21–23] and coating islets with ECM proteins prior to transplantation [15, 24]. However, the ECM alone may not be sufficient to promote survival. Improvement of ECM regeneration and revascularisation/angiogenesis is crucial for successful islet transplantation.

Here we apply a longitudinal and high-resolution imaging approach to investigate the dynamics of the pancreatic islet engraftment process up to 11 months after transplantation. For the current study, we used the anterior chamber of the mouse eye because it is well suited to study human and mouse pancreatic islet cell biology and revascularisation over time because of the transparency of the cornea, and its high potential to perform continuous repeated recordings on individual islet grafts [25–27].

## Methods

### Mice

B6-albino (B6(Cg)-*Tyr*^c-2J^/J; JAX000058) mice were purchased from The Jackson Laboratory (Bar Harbor, ME, USA). These mice were crossed with B6.129(Cg)-*Gt(ROSA)26Sor*^tm4^ mice (JAX007676) to generate B6(Cg)-*Tyr*^c-2J^-*Gt(ROSA)26Sor*^tm4^ (B6.*ROSA*-tomato) mice. NOD.(Cg)-*Gt(ROSA)26Sor*^tm4^ (NOD.*ROSA*-tomato) mice were generated by speed congenic backcrossing of the B6.129(Cg)-*Gt(ROSA)26Sor*^tm4^ mice to NOD mice for five generations (see electronic supplementary material [ESM] Methods, ESM Table 1). Finally, NOD.*ROSA*-tomato mice were crossed to NOD.*Rag2*^−/−^ [28] to generate NOD.*ROSA-*tomato.*Rag2*^−/−^. Tg.*Cspg4*-DsRed.T1.1Akik/J (JAX008241) mice were backcrossed to B6-albino to generate B6.(Cg)-*Tyr*^c-2J^-Tg.*Cspg4*-DsRed.T1.1Akik/J (B6.NG2-DsRed) mice. All animals were bred and maintained in a specific pathogen-free environment at the animal facilities at Lund University.

### Islet isolation, anterior eye chamber transplantation and in vivo imaging

Mouse islet isolation and transplantation to the anterior eye chamber of female 6–8 week old B6-albino or B6.*ROSA*-tomato mice were performed as previously described [26]. Human pancreatic islets of five nondiabetic brain-dead organ donors (ESM Table 2) were obtained from The Nordic Network for Islet Transplantation, through the Human Tissue Laboratory at Lund University Diabetes Center (Malmö, Sweden), cultured as described previously [29] and transplanted to the anterior eye chamber of female 6–8 week old NOD.*Rag2*^−/−^ or NOD.*ROSA*-tomato.*Rag2*^−/−^ mice. The Regional Ethics Committee in Lund, Sweden, approved the study according to the Act Concerning the Ethical Review of Research Involving Humans. In vivo imaging was performed as previously described [26, 27] using an upright laser scanning microscope (LSM 7 MP; Zeiss, Jena, Germany) equipped with a tunable Ti:sapphire laser (Spectra-Physics Mai Tai; Newport, CA, USA) and a long working distance 20×/1.0× water-dipping lens (Zeiss), specified in more detail in the ESM Methods. A 3D analysis of in vivo images of three to five randomly chosen islets per mouse eye was performed using Imaris 9.1 (Bitplane, Zurich, Switzerland) (ESM Fig. 1).

### Kidney transplantation model

Six to eight week old female recipient B6.*ROSA*-tomato mice were anaesthetised using inhalation anaesthesia (isoflurane; Schering-Plough, Kenilworth, NJ, USA) combined with analgesia using buprenorphine (0.15 mg/kg s.c.; RB Pharmaceuticals, Slough, UK). The islet suspension (30 μl) was injected between the capsule and renal parenchyma of the left kidney using a blunt cannula connected to a gastight syringe (Hamilton, Reno, NV, USA), as described previously [30].

### Tissue collection

Adult pancreases or isolated islets from 6–8 week old female mice and graft bearing kidneys (4 weeks after transplantation) and eyes were fixed in 4% paraformaldehyde/PBS (Sigma, St. Louis, MO, USA) for 30 min (islets) or 1.5 h (organs) on ice, equilibrated in 30% sucrose/PBS overnight at 4°C and cryopreserved in an optimum cutting temperature compound (VWR Scientific Products, Willard, OH, USA) at −80°C.

### Immunohistochemistry

Eight-micron cryosections were permeabalised and blocked in 10% goat serum in TRIS-buffered saline (TBS) 0.1% Triton X-100 for 1 h at room temperature and then incubated with primary antibodies overnight at 4°C (ESM Table 3). Appropriate secondary antibodies conjugated with Alexa 488/594/647 fluorophores from Life Technologies (Carlsbad, CA, USA) were applied for 1 h at room temperature (1:1000). Digital images of the cryosections mounted with fluorescence mounting medium (Dako, Glostrup, Denmark) were acquired with a Zeiss LSM 800 Airyscan confocal laser scanning microscope.

### Statistical analysis

Statistical analysis was performed using SPSS statistical software version 24 (IBM, Armonk, NY, USA) and Prism version 7 (Graphpad Software, San Diego, CA, USA). All the data are presented as median ± 95% CI. Friedman’s test was used to calculate the overall difference in time for repeated measurements and a Wilcoxon’s paired-sample test was used to compare all the different time points with the reference time point (1 week or 2 weeks). A Bonferroni correction was performed on paired-samples tests. We consequently used nonparametric methods since the sample sizes (i.e. the number of islets) associated with the tests were small (*n* ≤ 20) and thus assumptions regarding normal distribution of our data could not be assured.

## Results

### Revascularised islet grafts regenerate an ECM capsule similar to the peri-islet basement membrane

**Fig. 1:**
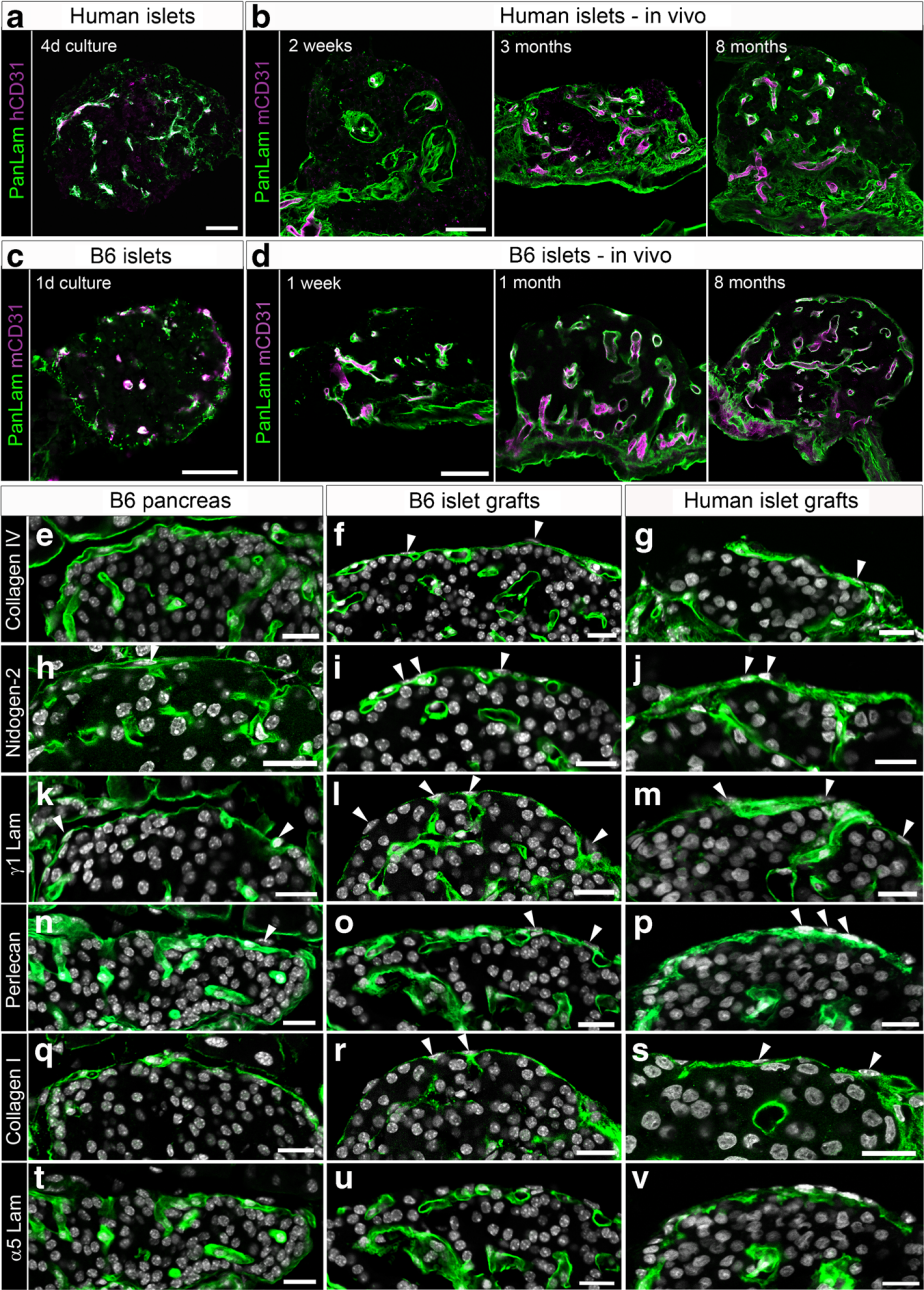
Distribution of endothelial cells and ECM proteins in islet basement membranes before and after transplantation into the anterior eye chamber of recipient mice. Cryosections of human (**a**, **b**) or B6-albino (B6) mouse islets (**c**, **d**) before (**a**, **c**) or after transplantation into the anterior eye chamber of B6-albino (**b**; representative of *n* = 3–5 mice, 4 islets per mouse) or NOD.*Rag2*^−/−^ recipient mice (**d**; representative of *n* = 10 mice, 3 islets per mouse) at the indicated time points were immunofluorescence labelled for pan-laminin (panLam, green) and human CD31 (**a**, hCD31) or mouse CD31 (**b–d**, mCD31, magenta). Co-localisation appears as white. (**e–v**) Distribution of the ECM proteins (in green) in the recipient B6-albino (B6) pancreas in situ (**e**, **h**, **k**, **n**, **q**, **t**) and in grafted mouse (**f**, **i**, **l**, **o**, **r**, **u**) or human islets (**g**, **j**, **m**, **p**, **s**, **v**) of the mouse recipients. Cryosections were immunolabelled for collagen IV (**e–g**), nidogen-2 (**h–j**), laminin chain γ1 (γ1 Lam; **k–m**), perlecan (**n–p**), collagen I (**q–s**) and laminin chain α5 (α5 Lam; **t–v**). DAPI staining (grey) indicates nucleated cells. Arrowheads indicate nucleated cells that were exterior or within the regenerated peri-islet ECM sheet. Scale bars, 50 μm (**a–d**) or 20 μm (**e–v**). d, day

The islet basement membrane is a sensitive biomarker of islet damage resulting from enzymatic isolation and of islet repair after transplantation. As predicted, human and mouse islets after isolation and prior to transplantation showed little or no laminin staining in the periphery of the islets, suggesting a complete degradation of the islet basement membrane with some residual intra-islet vascular basement membrane evident in the human islets 4 days after culture (Fig. 1a) and in mouse islets one day after culture (Fig. 1c). However, the isolated islets of both human and mouse origin retained their capillary network even after several days of culture (Fig. 1 b, d). To study how the ECM protein scaffold recovers upon transplantation, pancreatic islets of human organ donors or syngeneic mouse donors were transplanted into the anterior eye chamber of recipient mice [25]. Several weeks after transplantation, sections of islet grafts demonstrated that the surface of both human (Fig. 1b) and mouse islet grafts (Fig. 1d) was progressively ensheathed by a laminin-containing basement membrane. Similar to the peri-islet capsule of pancreatic islets in situ, revascularised islet grafts in the anterior eye chamber restored beta cell-matrix attachment with basement membrane components including collagen IV (Fig. 1e–g), nidogen-2 (Fig. 1h–j), laminins containing γ1 chains (Fig. 1k–m) and perlecan (Fig. 1n–p) as well as a common interstitial matrix component collagen I (Fig. 1 q–s). The peri-islet basement membrane is known to be biochemically distinct from vascular basement membranes [31–33] and in mice contains laminins such as γ1 chains (Fig. 1k–m) but not laminin α5 chains (Fig. 1t–u). In contrast, the vascular basement membranes contain both laminin α5 and γ1 chains. Interestingly, in the human peri-islet basement membrane, although all major components are the same as in the mouse, additional laminin α5 chains could be detected (Fig. 1v), a result consistent with findings from previous studies [33].

### Recruited recipient cells are responsible for the secretion of ECM proteins of the peri-islet-like basement membrane

**Fig. 2:**
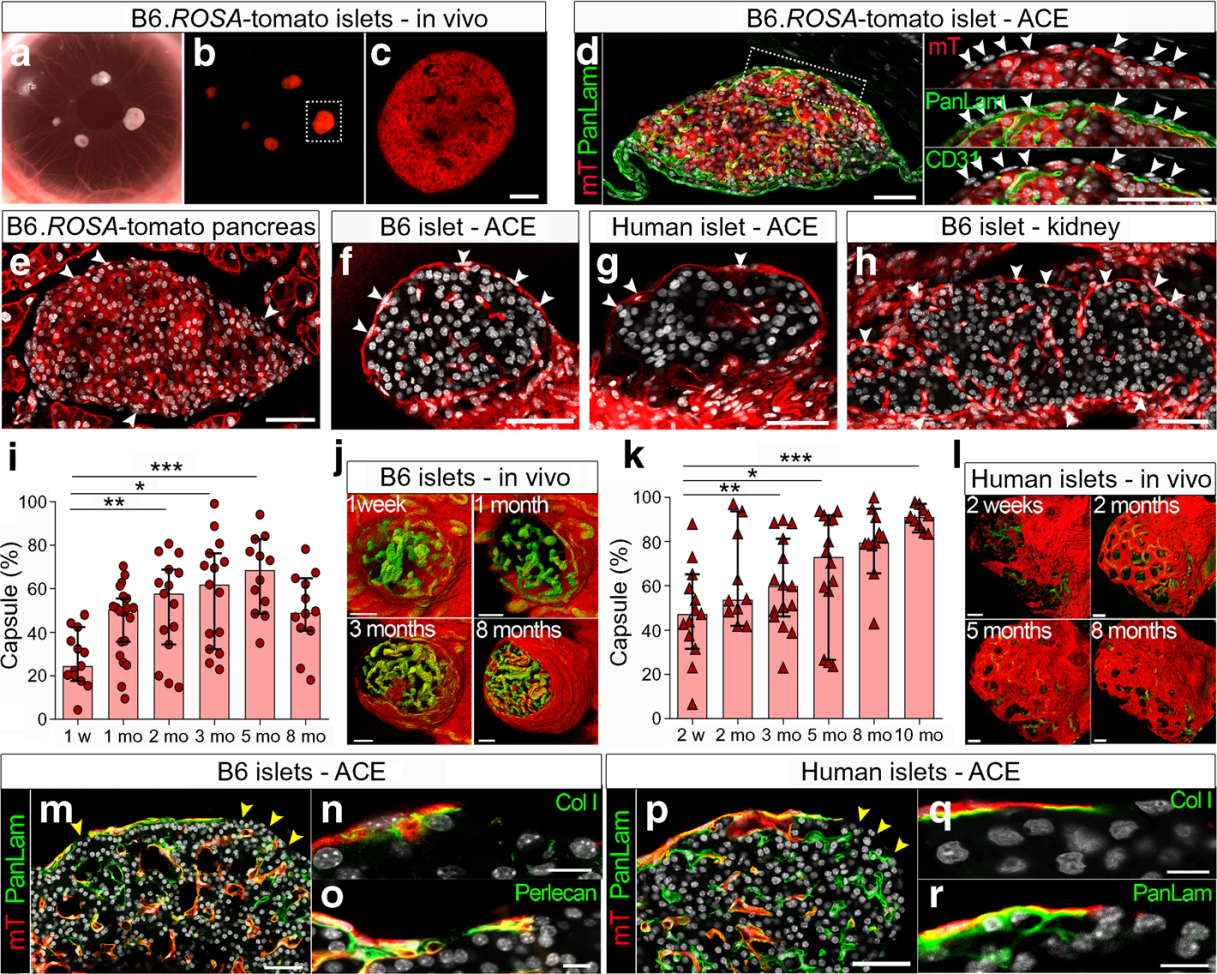
Transplanted islets of both mouse and human origin are progressively encapsulated by ECM-producing cells of recipient origin. B6.*ROSA*-tomato islets transplanted into the anterior chamber of the eye (ACE) of B6-albino recipient mice (**a–d**) or in situ in the pancreas (**e**), or B6-albino (B6) islets (**f**, **h**, **i**, **j**, **m–o**) or human islets (**g**, **k**, **l**, **p–r**) transplanted into the anterior eye chamber of B6.*ROSA*-tomato or NOD. *ROSA*-tomato.*Rag*2^−/−^ recipient mice, were imaged repeatedly for up to 10 months. (**a**) Photograph of the B6-albino recipient mouse eye transplanted with tomato islets engrafted on the iris. (**b**) Image obtained by a conventional fluorescence stereomicroscope and (**c**) optical section of a B6.*ROSA*-tomato islet, as indicated in (**b**), obtained by two-photon microscopy. (**d**) Cryosection of a B6.*ROSA*-tomato islet 2 months post transplantation stained for ECM marker pan-laminin (PanLam, green) or endothelial cell marker CD31 (green) and DAPI (grey). Membrane-targeted tomato fluorescence (mT) is used to visualise donor-specific islet cells (red). The white arrowheads indicate the cell nuclei of recipient origin (mT^−^ cells). (**e–h**) Cryosections of B6.*ROSA*-tomato pancreas (**e**), eyes of B6.*ROSA*-tomato (**f**) or NOD.*ROSA*-tomato.*Rag*2^−/−^ (**g**) recipient mice transplanted with B6-albino mouse islets (**f**) or human islets (**g**), or the kidney of B6.*ROSA*-tomato recipient mice bearing islet grafts (**h**). The white arrowheads indicate recipient cells (DAPI^+^ mT^+^) ensheathing the islet and surface of islet grafts. (**i**–**l**) Capsule surface analysis of mouse (**i**, **j**) or human (**k**, **l**) islet grafts. mT^+^ indicates recipient cells ensheathing the islet grafts at the indicated time points. 3D reconstructions are of vasculature (green) and capsule (red) of whole mouse (**j**) or human islet grafts (**l**) at the indicated time points post transplantation. (**i**, **k**) Values are given as median ± 95% CI for six mice (*n* = 30 mouse islets; **i**) and five mice (*n* = 18 human islets; **k**). The nonparametric Friedman test was used to test the overall differences and paired values were tested with a nonparametric Wilcoxon test with a Bonferroni correction. *p <0.1, **p <0.01, ***p <0.001. (**m–r**) Cryosections of eyes from B6.*ROSA*-tomato (**m**–**o**) or NOD.*ROSA*-tomato.*Rag*2^−/−^ (**p**–**r**) recipient mice transplanted with either B6-albino (**m–o**) or human islets (**p–r**) counterstained with nuclear marker DAPI (grey) and pan-laminin (**m**, **p**, **r**), collagen I (Col I; **n**, **q**) or perlecan (**o**) in green. The co-localisation of mT^+^ cells (red) and ECM (green) appears as yellow. Yellow arrowheads indicate the islet surface lacking mT^+^ cells and ECM proteins. Scale bars, 50 μm (**c–h**, **j**, **l**, **m**, **p**) or 10 μm (**n**, **o**, **q**, **r**). mo, month; w, week

In both human and mouse islet grafts, we noticed some cell nuclei outside the peripheral laminin staining suggesting that these cells constitute the origin of the repaired peri-islet-like basement membrane (Fig. 1e–s). To investigate the origin of these cells, we used a transplantation model in which either all cells of the donor islets (Fig. 2a–d) or all cells of the recipient mouse (Fig. 2e–l) would express a membrane-targeted tomato fluorescence protein (mT). B6(Cg)-*Tyr*^c-2J^-*Gt(ROSA)26Sor*^tm4^ (B6.*ROSA*-tomato) donor islet engrafted in the anterior eye chamber of B6-albino recipient mice showed a strong red fluorescence in vivo (Fig. 2a–c). Sections of the mT^+^ islet grafts counterstained with the basement membrane marker pan-laminin or the mouse endothelial marker CD31 (PECAM-1) indicated a peripheral mT^−^ cell layer of nonendothelial/nonvascular and recipient origin (Fig. 2d). To follow the fate of these cells, we transplanted nonlabelled mouse or human islets into the anterior eye chamber of B6.*ROSA*-tomato or NOD.(Cg)-*Gt(ROSA)26Sor*^tm4^-*Rag2*^−/−^ (NOD.*ROSA*-tomato.*Rag2*^−/−^) recipient mice (Fig. 2e–g). Although less frequently observed at the peri-islet capsule of B6.*ROSA*-tomato recipient mice in situ (Fig. 2e), spindle-shaped mT^+^ recipient cells could easily be detected encapsulating the transplanted mouse and human donor islet grafts (Fig. 2f, g). In addition, similar observations were made in the widely used renal islet transplantation model (Fig. 2h).

To follow the dynamics of mT^+^ recipient cells involved in the engraftment process, we repeatedly monitored islet grafts in vivo by two-photon microscopy (Fig. 2i–l). Continuous 3D imaging showed the progressive encapsulation of both human and mouse islets by mT^+^ recipient cells (median 68% of mouse vs 73% of human islet graft surfaces) until 5 months after transplantation (Fig. 2i, k). Later, the encapsulation process of syngeneic mouse islet grafts plateaued at 8 months, leaving larger islet surfaces uncovered (Fig. 2i, j). In contrast, from 2 months and up to 10 months, the outer host cell-derived capsule expanded and distributed evenly around human islets with a patchy dispersed pattern (Fig. 2k, l), remaining patchy throughout an extended imaging period of up to 11 months (ESM Fig. 2). To test if this patchiness could reflect the invagination of the peri-islet basement membrane together with ingrowing blood vessels, a more detailed 3D image segmentation analysis was performed, showing that the holes in the host cell-derived capsule were not directly connected to ingrowing blood vessels (ESM Fig. 3).

Cryosections of the islet grafts co-stained for ECM proteins demonstrate that only in areas where mT^+^ host cells have formed a single outer cell layer, a corresponding basement membrane-like structure is also formed, excluding remnant donor peri-islet basement membrane proteins as the possible reason (Fig. 2 m–r, ESM Fig. 4). But perhaps more important, the co-localisation of mT^+^ recipient cells with basement membrane proteins observed at 3–5 months post transplantation (Fig. 2m–r, ESM Fig. 4) or later (ESM Fig. 5) indicates that the host cells could be the main producers of the restored peri-islet-like basement membrane or possibly act as a scaffold for other ECM-producing cells.

### Identification of fibroblasts

**Fig. 3:**
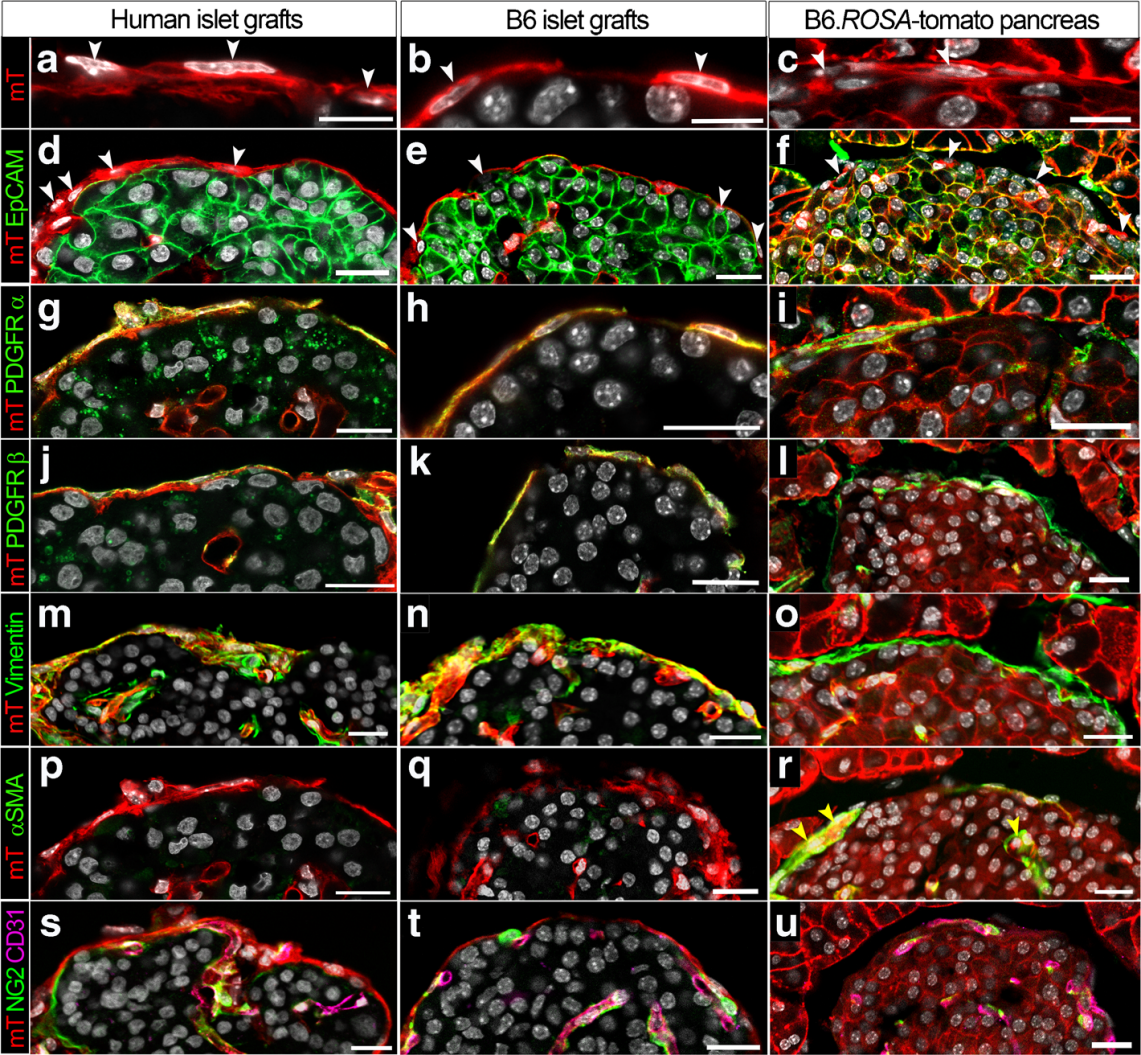
ECM-producing fibroblasts participate in the regeneration of the peri-islet-like basement membrane in transplanted islets and are associated with the peri-islet basement membrane in the pancreas. Cryosections of recipient NOD.*ROSA*-tomato.*Rag*2^−/−^ eyes with human islets (representative of *n* = 5 mice) or B6.*ROSA*-tomato eyes with B6-albino mouse islet grafts 3–5 months post transplantation (representative of *n* = 3–4 mice) or recipient B6.*ROSA*-tomato pancreas (representative of *n* = 3 mice) showing endogenous mT^+^ (red, **a–u**) and DAPI stain (grey) alone (**a**–**c**) or counterstained (in green) with epithelial marker EpCAM (**d–f**), PDGFRα (**g–i**), PDGFRβ (**j–l**), vimentin (**m–o**), αSMA (**p–r**) or NG2 (green) and CD31 (magenta) (**s–u**). Yellow indicates co-localisation. White arrowheads indicate nucleated mT^+^ cells in the ECM capsule and peri-islet basement membrane. Yellow arrowheads in (**r**) indicate αSMA^+^ blood vessels. See also ESM Fig. 6. Scale bars, 20 μm

We next studied the phenotype of the peri-islet-like basement membrane producing mT^+^ recipient cells in more detail using immunohistochemistry of recipient B6.*ROSA*-tomato pancreas or eyes transplanted with human or mouse islet grafts 3–5 months after transplantation (Fig. 3). The recipient origin of these cells could easily be discriminated from the donor islet parenchyma by the expression of the tomato/mT (Fig. 3a–c) and the absence of epithelial cell adhesion molecule (EpCAM) staining (Fig. 3d–f). Immunostaining with an anti-CD45 antibody demonstrated that these EpCAM^−^CD31^−^CD45^−^mT^+^ cells did not belong to the myeloid lineage (ESM Fig. 6). Further analysis showed that these cells co-expressed the fibroblast marker CD140a/platelet-derived growth factor receptor α (PDGFRα) (Fig. 3g–i) and podoplanin/gp38 (ESM Fig. 6), as well as the pericyte marker CD140b/PDGFRβ (Fig. 3j–l) and the intermediate filament marker vimentin (Fig. 3m–o) suggesting a mesenchymal origin of these cells. The lack of α-smooth muscle actin (αSMA) expression excludes them from being myofibroblasts (Fig. 3p–r). Furthermore, the cells were also found to lack the expression of mesenchymal stem/stromal cell (MSC) marker stem cell antigen 1 (Sca-1), Schwann cell marker glial fibrillary acidic protein (GFAP) and epithelial marker pan-cytokeratin (ESM Fig. 6). They were also negative for CD31 and the pericyte and vascular smooth muscle marker neuron-glial antigen 2 (NG2) (Fig. 3s–u) excluding them from being a population of mature pericytes. Finally, histological analyses allowed us to define a generic cell surface antigen profile of an ECM-producing fibroblast-like recipient cell in the peri-islet-like basement membrane of islet grafts as follows: CD31^−^, CD45^−^, NG2^−^, EpCAM^−^, E-Caherin^−^, Sca-1^−^, PDGFRα^+^ and PDGFRβ^+^ in combination with a cytoplasmic expression profile of αSMA^−^, GFAP^−^, gp38^+^ and vimentin^+^. This cell population could also be identified in association with the peri-islet capsule in situ (Fig. 3c–u, ESM Fig. 6).

**Fig. 4:**
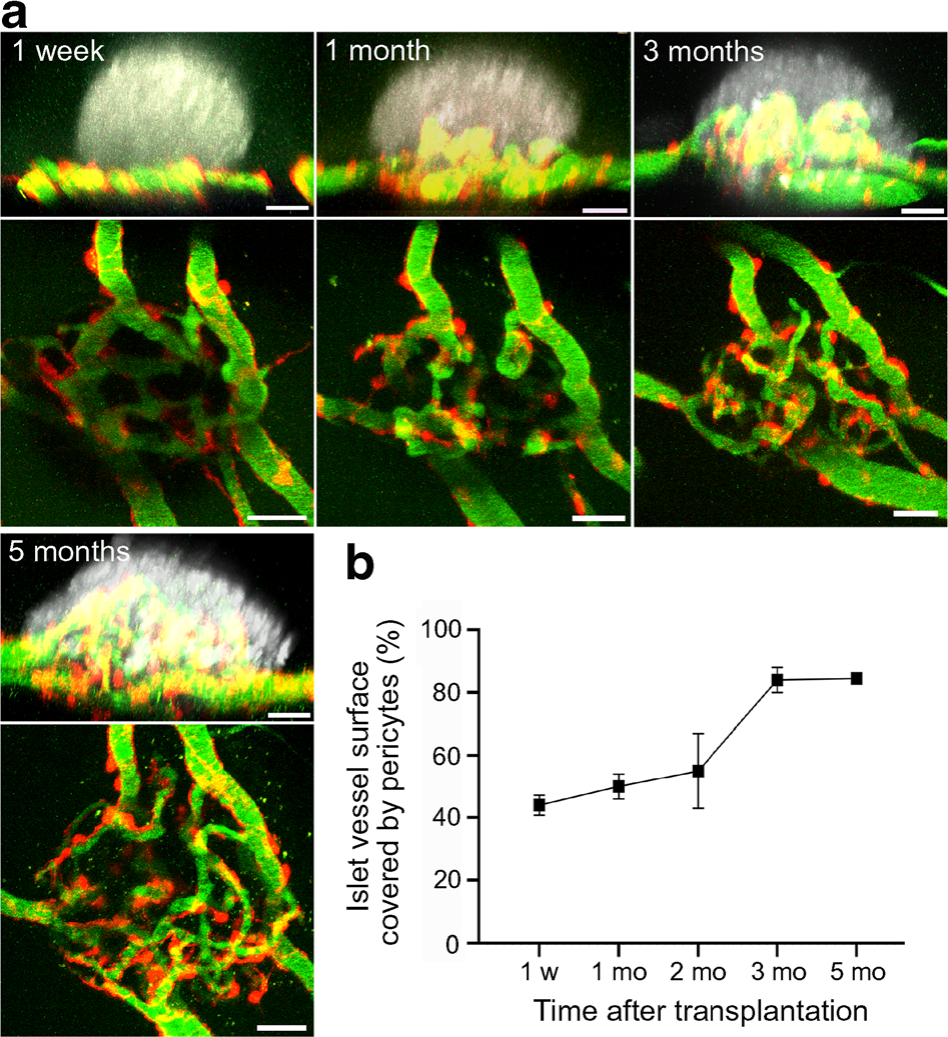
NG2^+^ pericytes of the host are progressively migrating and accumulating along the vessels of islet grafts. B6-albino islets were transplanted into the anterior eye chamber of B6.NG2-DsRed mice (*n* = 3) and imaged repeatedly for up to 5 months post transplantation. (**a**) Merged vertical maximum intensity projections (MIPs) of the islet structure based on backscatter light and NG2^+^ pericytes (red) and vasculature (green) (upper images) or horizontal MIPs from NG2^+^ pericytes and vasculature alone (lower images) of one representative islet graft imaged repetitively at the indicated time points. (**b**) Quantification of the proportion of pericytes covering the total islet vessel surface. Scale bars, 50 μm. mo, month; w, week

Because both fibroblasts and pericytes are spindle-shaped ECM-producing cells with mesenchymal origin, hence possibly showing some degree of plasticity [34, 35], we next analysed transplanted islet grafts in the anterior eye chamber of B6.NG2-DsRed reporter mice. As illustrated in Fig. 4, despite numerous NG2^+^ pericytes progressively accumulating in association with vasculature inside the islet grafts (Fig. 4a), NG2^+^ cells were only rarely detected on the islet surface. The proportion of the abluminal islet vessel surface, which is covered by pericytes, ranged between 44% at 2 weeks post transplantation to 85% at 5 months post transplantation (Fig. 4b).

### Transplanted human islets retain their vessel network density when transplanted into a nonhuman host

**Fig. 5:**
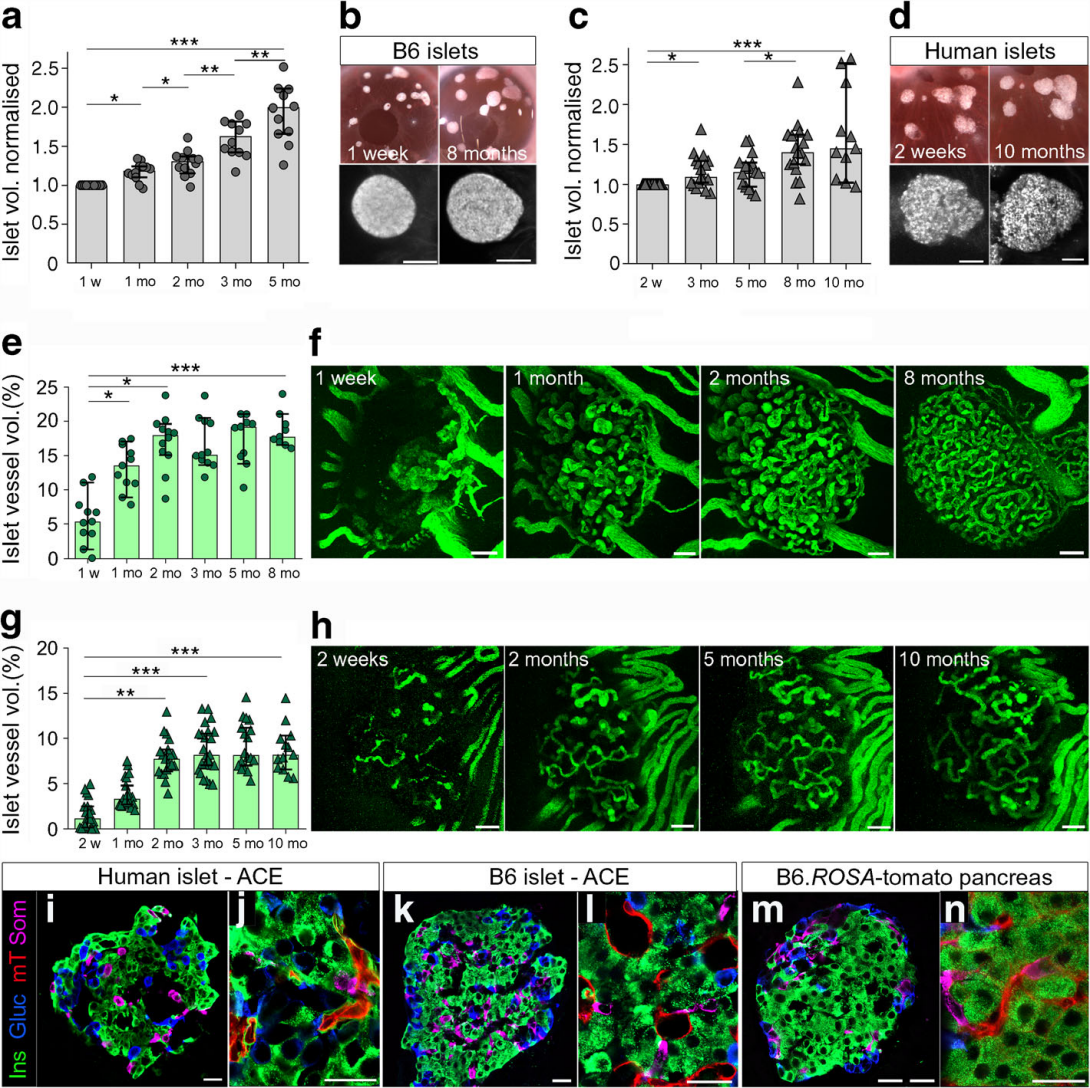
Structure, vessel network and cellular composition of mouse and human islets after transplantation in the anterior eye chamber graft site. Islet volume analysis (**a–d**) and vessel volume analysis (**e–h**) in transplanted mouse (**a**, **b**, **e**, **f**) or human islet grafts (**c**, **d**, **g**, **h**) imaged repeatedly by two-photon microscopy for up to 10 months post transplantation. Islet volume analysis for mouse islets (**a**) or human islets (**c**) is based on backscatter light and normalised to the initial volume in week 1 (**a**) or week 2 (**c**). Representative images show photographs of the recipient mouse eye transplanted with islets engrafted on the iris and maximum intensity projections (MIPs) of backscatter light (grey) of an islet at week 1 and month 8 (**b**) and week 2 and month 10 (**d**). A vessel volume analysis of the transplanted mouse (**e–f**) and human islets (**g–h**) in vivo, showing islet vessel volume fraction (**e**, **g**) and MIPs of vessels (**f**, **h**) of individual islets at indicated time points. (**a**, **c**, **e**, **g**) Values are given as median ± 95% CI for three mice (*n* = 13 mouse islets; 4–5 islets per mouse; **a**, **e**) and 13 mice (*n* = 38 human islets; 3–6 islets per mouse; **c**, **g**). The nonparametric Friedman test was used to test the overall differences and paired values were tested with a nonparametric Wilcoxon test with a Bonferroni correction. *p <0.1, **p <0.01, ***p <0.001. Representative immunofluorescence images of human (**i**, **j**; *n* = 4 mice, 3–4 islets) or B6 mouse islet grafts (**k**, **l**; *n* = 3 mice, 3–5 islets) 5 months after transplantation, or B6.*ROSA*-tomato mouse pancreas (**m**, **n**; *n* = 3 mice) stained with antibodies specific for insulin (Ins; green), glucagon (Gluc, blue), and somatostatin (Som, magenta). Endogenous mT^+^ visualising vasculature is shown in red (**j**, **l**, **n**). Scale bars, 100 μm (**b**, **d**), 50 μm (**f**, **h**), or 20 μm (**i–n**). ACE, anterior chamber of the eye; mo, month; vol, volume; w, week

Individual human or mouse islet grafts were repeatedly imaged in vivo by confocal and two-photon microscopy to record detailed structural and vascular changes over a period of up to 10 months. In agreement with previous data [25], mouse islets expanded throughout the study (Fig. 5a, b), reaching a twofold increase in median volume already at 5 months post transplantation. Human islets expanded significantly less reaching a median 1.45-fold increase in volume after 10 months post transplantation (Fig. 5c, d).

Both human and mouse islets displayed a similar initial vessel density increase reaching a plateau at about 2 months post transplantation (Fig. 5e–h). Although mouse islet grafts reached a vessel density of about 18% (Fig. 5e, f), the newly formed vessel network of human islet grafts reached a density of only 9% (Fig. 5g, h). The pancreatic islet grafts showed an intact anatomical organisation in a species-specific manner, with beta cells scattered over the entire human islet volume (Fig. 5i) and most beta-, alpha-, and delta cells closely but randomly associated with the mT^+^ vascular cells of the mouse recipient (Fig. 5j), similar to human islets in situ [36]. Despite the clear segregation of cell types to different regions of the mouse islet (Fig. 5 k), all endocrine cell types examined in mouse islets were randomly associated with blood vessels without the previously proposed clustering of beta cells (Fig. 5l), similar to mouse islets in situ (Fig. 5m, n). This well-established difference from the anatomical organisation is also illustrated in the homogenous vs nonhomogeneous tissue structure revealed in backscatter images of mouse (Fig. 5b) or human islets (Fig. 5d).

### Role of intra-islet endothelial cells differs in syngeneic and interspecies islet transplantations

**Fig. 6:**
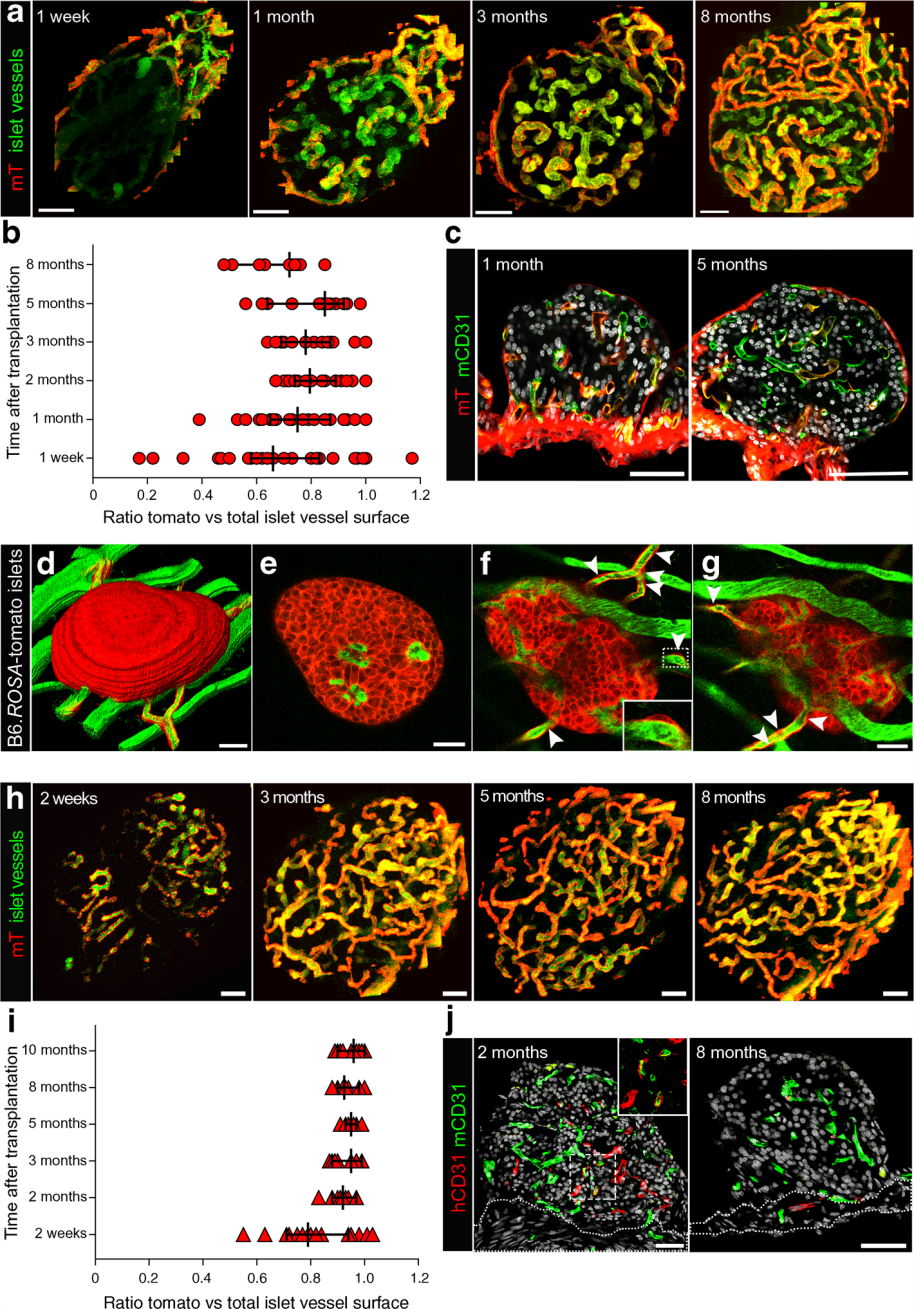
Contribution of donor and mT^+^ recipient cells in the islet revascularisation of islet grafts. B6-albino islets transplanted into the anterior eye chamber of B6.*ROSA*-tomato recipient mice (**a–c**; *n* = 7) or B6.*ROSA*-tomato islets, transplanted into the anterior eye chamber of B6-albino recipients (**d–g**; representative of *n* = 3 mice) or human islets transplanted into the anterior eye chamber of NOD.*ROSA*-tomato.*Rag2*^−/−^ (**h–i**; *n* = 10 mice) or NOD.*Rag2*^−/−^ (**j**; *n* = 6 mice) recipient mice, were imaged repeatedly for up to 8 months (**a–c**) or 3 months (**d–g**) or 10 months (**h–i**). Shown here are the maximum intensity projections (MIPs) of islet vasculature (green) and islet vasculature associated mT^+^ recipient cells (red) (**a**, **h**) and the ratio of mT^+^ recipient cell vs total vessel surface based on a surface rendering of the chimeric islet vessel network (**b**, **i**). Yellow (merge of red and green) indicates vessels of recipient origin and green the remnant donor origin (**a**, **h**). For visualisation and quantification of islet vasculature, image stacks were processed excluding the signal from the iris and the mT^+^ capsule (see ESM Fig. 1). (**c**) Cryosections of islet grafts at the indicated time points post transplantation showing endogenous mT^+^ (red) counterstained with endothelial CD31 marker (green) and DAPI (grey) (*n* = 3 mice each, 6 islets per time point). 3D rendering (**d**) or optical sections (**e–g**) of an mT^+^ labelled donor islet 1 week post transplantation. The arrowheads in (**f**, **g**) indicate mT^+^ donor cells (red) migrating out of the islet along the vessels of the iris of the recipient (green). The location of the magnified image in (**f**) is indicated by a dashed line. (**j**) The cryosections of human islet grafts at the indicated time points post transplantation immunolabelled with human endothelial CD31 marker (hCD31, red) and mouse endothelial CD31 marker (mCD31, green); DAPI staining is shown in grey; the dotted lines indicate the iris area. The location of the magnified image is indicated by a dashed line (*n* = 5 islets per time point). (**a**, **b**, **h**, **i**) Representative images and values given as median ± 95% CI for six mice (*n* = 32 mouse islets; **b**) and five mice (*n* = 22 human islets; **i**). See also ESM Video 1 relating to mouse islet graft at 8 months post transplantation (**a**) and ESM Video 2, relating to human islet graft 8 months post transplantation (**h**). Scale bars, 50 μm. mo, month; vasc., vascular; w, week

Islets engrafted in the anterior eye chamber of B6.*ROSA*-tomato or NOD.*ROSA*-tomato.*Rag*2^−/−^ recipient mice allows long-term monitoring of mT^+^ recipient cells and their contribution to the revascularisation process over several months (Fig. 6). In the transplanted mouse islets, a functional vessel network was established between 1 and 2 months after transplantation (Fig. 1e). Figure 6a illustrates the immediate revascularisation through an initial recruitment of mT^+^ recipient cells generating a chimeric endothelium that included vessel sections of either donor or recipient origin or chimeric vessel sections of both donor intra-islet cells and recipient derived cells, a result consistent with previous studies [30, 37]. Over time, the representation of mT^+^ recipient cells increased gradually ranging between 67% and 100% recipient origin (median ratio 0.79 at 2 months post transplantation) in some vessels (Fig. 6b, ESM Video 1). However, the chimeric pattern of the vascular endothelium showed dynamic changes over time, and this was accompanied by a gradual decrease of mT^+^ recipient cells in later time points. Cryosections at several time points during the revascularisation process counterstained for the mouse endothelial cell marker CD31 confirmed the chimerism of donor and recipient endothelial cells in vessels of mouse islet grafts (Fig. 6c).

The progressive replacement of the endothelial cells of donor origin by cells of a recipient origin is a result of cell migration but could potentially also depend on proliferation of cells in situ. Similarly, the gradual disappearance of recipient cells could be because of cell death and/or cell migration out of the islet transplant. To test for the latter hypothesis, we transplanted mT^+^ labelled islets into nonlabelled recipient mice. Occasionally we observed mT^+^ cells migrating from the mT^+^ labelled islet grafts along the blood vessels into the iris of B6-albino recipient mice (Fig. 6d–g). This data supported the notion that the disappearance of donor cells in the engrafted islet was partly a result of cellular migration.

In contrast, human islet grafts did not show purely non-tomato/donor vessel sections at any time point of imaging during the current study (Fig. 6h). Despite a chimeric tendency observed at early time points (median ratio of 0.79 at week 2, Fig. 6i), indicating that human endothelial cells might be present, from 2 months after transplantation, vessels were found to be covered mainly by mT^+^ recipient cells (median ratio 0.92, Fig. 6h, i, ESM Video 2). Cryosections of human islet grafts (NOD.*Rag2*^−/−^ recipients) stained with species-specific antibodies to the endothelium-specific marker CD31 demonstrate that human intra-islet endothelial cells survive several days of culture prior to transplantation (Fig. 1a) and even for a couple of months after transplantation as part of vascular-like structures within the revascularised islet grafts (Fig. 6j). However, human endothelial cells disappeared at later time points in established human grafts. Frequent detection of human CD31 positive cells in the iris vasculature of the recipient (Fig. 6j) indicates that at least a part of them migrated out of the graft as seen for transplanted mT^+^ mouse islets (Fig. 6d–g) and as reported previously for renal islet grafts [30].

## Discussion

In the present study we applied 3D longitudinal in vivo imaging of human pancreatic islets transplanted into the anterior eye chamber of recipient mice to reveal the involvement of a motile fibroblast population in the reconstitution of the peri-islet basement membrane of transplanted pancreatic islets. The conditions studied here represent a transplantation situation in which the peri-islet basement membrane has been severely damaged or even removed. Following transplantation, we could trace the population of fibroblast-like cells migrating from the host to the periphery of syngeneic or xenogeneic transplanted islets, aiding in the formation of a basement membrane-like structure. In this transplantation situation the fibroblast-like stromal cells appear to take over as main producers of ECM as they reconstitute the peri-islet-like basement membrane. This was observed in syngeneic mouse-to-mouse islet transplantations and was also observed in interspecies human to mouse transplantations. To the best of our knowledge, no study has specifically visualised the association of an ECM-producing cell type with the peri-islet basement membrane. In the current study, the recruited cells could be identified with a surface marker profile as CD31^−^, CD45^−^, NG2^−^, EpCAM^−^, E-Caherin^−^, Sca-1^−^, PDGFRα^+^ and PDGFRβ^+^. In combination with the expression of vimentin and gp38—but not αSMA cytoplasmic protein— this excluded them from being myofibroblasts.

The recruited fibroblasts were found to produce ECM proteins such as collagen IV, nidogen-2, laminin γ1, perlecan and fibrillary collagen I as well as laminin α5 chains specifically in human islet grafts [32]. This agrees with the major peri-islet basement membrane components that have been reported for the pancreas. Based on studies with endpoint histological analysis, the re-establishment of basement membrane matrix proteins has been observed for mouse [38] and human [39] islet kidney transplantations. One particular study showed that the peri-islet deposition of basement membrane matrix proteins coincided with the localisation of PECAM-1-positive vascular endothelial cells (VECs) that had migrated to the periphery of the syngeneic islets. This is possibly because of the nature of the graft site. Host VECs that accumulate at the renal graft site stroma to initiate the revascularisation process are then resolved when the VECs migrated into the islets to form the intra-islet vascular endothelium. Only occasionally could we observe CD31^+^ VECs in close proximity to the islet periphery, usually in association with NG2^+^ pericytes. However, CD31^+^ VECs were mainly found to be involved in the revascularisation process from the iris.

The recruited fibroblasts accumulated in large quantities and formed a dense fibroblast network of the islet graft fibroblast capsule. In human islet grafts the fibroblasts progressively formed capsules covering the islet graft surfaces almost completely (up to median 91% [95% CI 84, 97] at 10 months post transplantation) leaving only small openings that also remained during the time of observation up to 11 months. In contrast, the encapsulation of mouse islet grafts was progressive but incomplete during the time of observation (median 49% [95% CI 40, 64] at 8 months post transplantation) with larger islet surfaces lacking mT^+^ recipient cells. Given that recruited fibroblasts were found to co-localise with ECM proteins, we examined those gaps in the islet capsule in more detail. Islet graft surfaces that were not covered by mT^+^ fibroblasts also lacked peripheral islet ECM proteins. The reason that those larger peripheral areas were lacking a basement membrane remains unclear but the different islet preparation and preculture of human and mouse islets could cause an altered density of ECM ligands contributing to a difference in fibroblast movement and encapsulation. Moreover, while a direct correlation of individual ECM components within the encapsulation could not be evidenced, we speculate that the well-established differences in the interspecies specialisation of islet architecture and islet innervation between mice and humans [40, 41] could contribute to these discrepancies. In this context, our understanding of how important the formation of the peri-islet membrane is in the innervation of the grafts remains largely unknown.

Immediately after transplantation, the islet depends on the diffusion of oxygen and nutrients from the surrounding microenvironment for its survival and function. We found that endothelial cells from the transplant recipient are recruited into the islet graft, already creating new islet vessel networks within several days and reaching islet vessel densities comparable to islets in situ after 2–3 months post transplantation. The newly formed vessel network needs to be stabilised through the recruitment of supporting cells such as pericytes [42]. Pericytes are peri-endothelial cells that cover capillaries and other micro vessels with adaptive plasticity as shown in response to islet injury [43] or renal islet transplantation in mice [44]. We show that NG2^+^ pericytes are actively involved in the revascularisation process, progressively accumulating in perivascular domains in transplanted islets up to 5 months after transplantation. Mature pericytes were only rarely detected in the periphery of the islet without being associated with vasculature, suggesting that they were not involved in the reconstitution of the peri-islet basement membrane.

Despite the similar initial vessel density increase in syngeneic and interspecies transplantations, the mouse islet grafts reached a vessel density of about 17%, while the newly formed vessel network of human islet grafts reached a density of only 9%. This is in agreement with the substantially lower capillary density found in human vs mouse endocrine pancreas [30] and in accordance with the vessel densities of islets previously recorded in situ in mouse pancreas and human live pancreas sections [45]. Several studies have reported that transplanted islets display reduced vessel density compared with pancreatic islets in situ [46, 47]. The involvement of intra-islet and recipient endothelial cells in human islet graft revascularisation is, however, still not completely understood and has not been studied in a longitudinal manner. In the current study, we followed transplanted islets by repetitive imaging for up to 11 months post transplantation. In our model system, endothelial cells from the recipient were the main contributor to the revascularisation of human islets. Initially, intra-islet endothelial cells constituted an alternative vascular source that exists in isolated islets and can account for up to 20% of the endothelial cells lining functional capillaries within the first weeks of revascularised xenogeneic human grafts. However, fully revascularised human islet grafts contained vessels of a purely mouse recipient origin. Human endothelial cells rapidly disappeared and at least parts of them migrated out of the graft. We conclude that even though recipient endothelial cells are mainly involved in the revascularisation process, it is cues produced by the donor islet that determine the structure and density of the vessel network, possibly by providing complex matrices and preformed paths that the recipient cells may navigate along.

In summary, our data provides evidence of a fibroblast population as the main organiser of the ECM encapsulation of transplanted islets of Langerhans and of islet-derived factors acting as cues for the architecture of the revascularisation of these islets. This may have implications for our understanding of long-term graft rejection and for the design of novel strategies to interfere with this process.

## Electronic supplementary material

ESM (PDF 2282 kb)

ESM Video 1 Contribution of donor and mT^+^ recipient cells in the islet revascularisation of a syngeneic mouse islet graft8 months post transplantation (relating to Figure 6a). Three dimensional rendering showing vessel volume in green (by i.v. injection of imaging agent) and mT^+^ recipient cells visualised by membrane-targeted and constitutively expressed membrane targeted Tomato in red. For visualisation purpose the total mT^+^ cell population was separated into mT^+^ cells of the islet capsule (identified as fibroblasts) and vessel associated mT^+^ recipient cells. Shown is a chimeric pattern of green and yellow (merge of red and green) vessel surfaces. Green vessel surface indicates donor origin and red or yellow vessel surface indicates recipient cell origin. (MP4 10,348 kb)

ESM Video 2 Contribution of donor and mT^+^ recipient cells in the islet revascularisation of a human islet graft8 months post transplantation (relating to Figure 6). Three dimensional rendering showing vasculature in green and mT^+^ recipient cells in red. For visualisation purpose the total mT^+^ cell population was separated into mT^+^ cells of the islet capsule (identified as fibroblasts) and vessel associated mT^+^ recipient cells. Red or yellow (merge of red and green) vessel surface indicates recipient cell origin. (MP4 11,200 kb)

## Data Availability

The datasets generated and/or analysed during the current study are available from the corresponding author upon reasonable request.

## Abbreviations

ECM: Extracellular matrix
EpCAM: Epithelial cell adhesion molecule
GFAP: Glial fibrillary acidic protein
mT: Membrane-targeted tomato fluorescence protein
NG2: Neuron-glial antigen 2
PDGFR: Platelet-derived growth factor receptor
Sca-1: Stem cell antigen 1
SMA: Smooth muscle actin
VEC: Vascular endothelial cell

## References

[1] Geddes J, Schopman JE, Zammitt NN, Frier BM (2008). Prevalence of impaired awareness of hypoglycaemia in adults with type 1 diabetes. Diabet Med.

[2] McCrimmon RJ, Sherwin RS (2010). Hypoglycemia in type 1 diabetes. Diabetes.

[3] Eich T, Eriksson O, Lundgren T, Nordic Network for Clinical Islet T (2007). Visualization of early engraftment in clinical islet transplantation by positron-emission tomography. N Engl J Med.

[4] Nanji SA, Shapiro AM (2006). Advances in pancreatic islet transplantation in humans. Diabetes Obes Metab.

[5] Pepper AR, Gala-Lopez B, Ziff O, Shapiro AM (2013). Revascularization of transplanted pancreatic islets and role of the transplantation site. Clin Dev Immunol.

[6] Lau J, Henriksnas J, Svensson J, Carlsson PO (2009). Oxygenation of islets and its role in transplantation. Curr Opin Organ Transplant.

[7] Wang RN, Rosenberg L (1999). Maintenance of beta-cell function and survival following islet isolation requires re-establishment of the islet-matrix relationship. J Endocrinol.

[8] Llacua LA, Faas MM, de Vos P (2018). Extracellular matrix molecules and their potential contribution to the function of transplanted pancreatic islets. Diabetologia.

[9] Smink AM, de Vos P (2018). Therapeutic strategies for modulating the extracellular matrix to improve pancreatic islet function and survival after transplantation. Curr Diab Rep.

[10] Pinkse GG, Bouwman WP, Jiawan-Lalai R, Terpstra OT, Bruijn JA, de Heer E (2006). Integrin signaling via RGD peptides and anti-beta1 antibodies confers resistance to apoptosis in islets of Langerhans. Diabetes.

[11] Rozario T, DeSimone DW (2010). The extracellular matrix in development and morphogenesis: a dynamic view. Dev Biol.

[12] Ramos Gde O, Bernardi L, Lauxen I, Sant'Ana Filho M, Horwitz AR, Lamers ML (2016). Fibronectin modulates cell adhesion and signaling to promote single cell migration of highly invasive oral squamous cell carcinoma. PLoS One.

[13] Aamodt KI, Powers AC (2017). Signals in the pancreatic islet microenvironment influence beta-cell proliferation. Diabetes Obes Metab.

[14] Bogdani M, Korpos E, Simeonovic CJ, Parish CR, Sorokin L, Wight TN (2014). Extracellular matrix components in the pathogenesis of type 1 diabetes. Curr Diab Rep.

[15] Llacua A, de Haan BJ, Smink SA, de Vos P (2016). Extracellular matrix components supporting human islet function in alginate-based immunoprotective microcapsules for treatment of diabetes. J Biomed Mater Res A.

[16] Morini S, Braun M, Onori P (2006). Morphological changes of isolated rat pancreatic islets: a structural, ultrastructural and morphometric study. J Anat.

[17] Wang RN, Paraskevas S, Rosenberg L (1999). Characterization of integrin expression in islets isolated from hamster, canine, porcine and human pancreas. J Histochem Cytochem.

[18] Stendahl JC, Kaufman DB, Stupp SI (2009). Extracellular matrix in pancreatic islets: relevance to scaffold design and transplantation. Cell Transplant.

[19] Chen CS, Mrksich M, Huang S, Whitesides GM, Ingber DE (1997). Geometric control of cell life and death. Science.

[20] Frisch SM, Francis H (1994). Disruption of epithelial cell-matrix interactions induces apoptosis. J Cell Biol.

[21] Lucas-Clerc C, Massart C, Campion JP, Launois B, Nicol M (1993). Long-term culture of human pancreatic islets in an extracellular matrix: morphological and metabolic effects. Mol Cell Endocrinol.

[22] Montesano R, Mouron P, Amherdt M, Orci L (1983). Collagen matrix promotes reorganization of pancreatic endocrine cell monolayers into islet-like organoids. J Cell Biol.

[23] Sigmundsson K, Ojala JRM, Ohman MK (2018). Culturing functional pancreatic islets on alpha5-laminins and curative transplantation to diabetic mice. Matrix Biol.

[24] Salvay DM, Rives CB, Zhang X (2008). Extracellular matrix protein-coated scaffolds promote the reversal of diabetes after extrahepatic islet transplantation. Transplantation.

[25] Berclaz C, Schmidt-Christensen A, Szlag D (2016). Longitudinal three-dimensional visualisation of autoimmune diabetes by functional optical coherence imaging. Diabetologia.

[26] Schmidt-Christensen A, Hansen L, Ilegems E (2013). Imaging dynamics of CD11c(+) cells and Foxp3(+) cells in progressive autoimmune insulitis in the NOD mouse model of type 1 diabetes. Diabetologia.

[27] Speier S, Nyqvist D, Cabrera O (2008). Noninvasive in vivo imaging of pancreatic islet cell biology. Nat Med.

[28] Soderstrom I, Bergman ML, Colucci F, Lejon K, Bergqvist I, Holmberg D (1996). Establishment and characterization of RAG-2 deficient non-obese diabetic mice. Scand J Immunol.

[29] Goto M, Eich TM, Felldin M (2004). Refinement of the automated method for human islet isolation and presentation of a closed system for in vitro islet culture. Transplantation.

[30] Brissova M, Fowler M, Wiebe P (2004). Intraislet endothelial cells contribute to revascularization of transplanted pancreatic islets. Diabetes.

[31] Irving-Rodgers HF, Ziolkowski AF, Parish CR (2008). Molecular composition of the peri-islet basement membrane in NOD mice: a barrier against destructive insulitis. Diabetologia.

[32] Korpos E, Kadri N, Kappelhoff R (2013). The peri-islet basement membrane, a barrier to infiltrating leukocytes in type 1 diabetes in mouse and human. Diabetes.

[33] Virtanen I, Banerjee M, Palgi J (2008). Blood vessels of human islets of Langerhans are surrounded by a double basement membrane. Diabetologia.

[34] Armulik A, Genove G, Betsholtz C (2011). Pericytes: developmental, physiological, and pathological perspectives, problems, and promises. Dev Cell.

[35] Guimaraes-Camboa N, Cattaneo P, Sun Y (2017). Pericytes of multiple organs do not behave as mesenchymal stem cells in vivo. Cell Stem Cell.

[36] Brissova M, Fowler MJ, Nicholson WE (2005). Assessment of human pancreatic islet architecture and composition by laser scanning confocal microscopy. J Histochem Cytochem.

[37] Nyqvist D, Speier S, Rodriguez-Diaz R (2011). Donor islet endothelial cells in pancreatic islet revascularization. Diabetes.

[38] Irving-Rodgers HF, Choong FJ, Hummitzsch K, Parish CR, Rodgers RJ, Simeonovic CJ (2014). Pancreatic islet basement membrane loss and remodeling after mouse islet isolation and transplantation: impact for allograft rejection. Cell Transplant.

[39] Lavallard V, Armanet M, Parnaud G (2016). Cell rearrangement in transplanted human islets. FASEB J.

[40] Dolensek J, Rupnik MS, Stozer A (2015). Structural similarities and differences between the human and the mouse pancreas. Islets.

[41] Taborsky GJ (2011). Islets have a lot of nerve! Or do they?. Cell Metab.

[42] Jain RK (2003). Molecular regulation of vessel maturation. Nat Med.

[43] Tang SC, Chiu YC, Hsu CT, Peng SJ, Fu YY (2013). Plasticity of Schwann cells and pericytes in response to islet injury in mice. Diabetologia.

[44] Juang JH, Kuo CH, Peng SJ, Tang SC (2015). 3-D imaging reveals participation of donor islet Schwann cells and pericytes in islet transplantation and graft neurovascular regeneration. EBioMedicine.

[45] Cohrs CM, Chen C, Jahn SR (2017). Vessel network architecture of adult human islets promotes distinct cell-cell interactions in situ and is altered after transplantation. Endocrinology.

[46] Carlsson PO, Palm F, Andersson A, Liss P (2001). Markedly decreased oxygen tension in transplanted rat pancreatic islets irrespective of the implantation site. Diabetes.

[47] Mattsson G, Jansson L, Carlsson PO (2002). Decreased vascular density in mouse pancreatic islets after transplantation. Diabetes.

