# Supplementary material for: Recruited fibroblasts reconstitute the peri-islet membrane: a longitudinal imaging study of human islet grafting and revascularisation
Source: Diabetologia. 2019 Nov 7;63(1):137–48. doi: 10.1007/s00125-019-05018-1 (PMC6890581; doi:10.1007/s00125-019-05018-1)
Supplement: Supplementary file 1 — (PDF 2282 kb) [file 125_2019_5018_MOESM1_ESM.pdf]

ESM Data to Nilsson J et al:

ESM methods

In vivo imaging

Recipient mice were anesthetized using inhalation anaesthesia (isoflurane; Schering-Plough, Kenilworth, NJ) and administered buprenorphine (0.15 mg/kg; RB Pharmaceuticals, Slough, UK) subcutaneously. Animals were fixated and kept on a heating pad during the imaging procedure. Repetitive *in vivo* imaging was performed at indicated time points using a fluorescence stereoscopic microscope (Multizoom AZ100; Nikon, Tokyo, Japan) and for confocal and two-photon imaging, using an upright laser scanning microscope (LSM 7 MP; Zeiss, Jena, Germany) equipped with a tunable Ti:sapphire laser (Spectra-Physics Mai Tai; Newport, CA) and a long working distance 20x/1.0x water-dipping lens (Zeiss, Jena, Germany). The total volume of transplanted islets was assessed by detection of 633 nm laser backscatter. The vessels were visualized by injecting 70-kDa- FITC-Dextran (100 µl of 2.5 mg/ml; (Thermo Fisher Scientific, Darmstadt, Germany) in PBS or Angiosense 680 (100 µl; Perkin Elmer, Waltham, MA) into the tail vein. We excited tomato and FITC or angiosense 680 at 900 nm and collected the emission light onto two or three nondescanned detectors using a dicronic mirror (LBF760) and emission filters (LP680 and BP690-730 for angiosense; LP555 and BP500-550 for FITC; mirror and BP 565-610 for tomato). Z-stacks were acquired with a step size of 2 µm for vessel/capsule and 3µm for backscatter signal. Quantitative analysis was carried out on three to five randomly selected islets per mouse.

Speed congenics

NOD.(Cg)-*Gt(ROSA)26Sor*^{tm4} (NOD.*ROSA*-tomato) mice were generated by speed congenic backcrossing of the B6.129(Cg)-*Gt(ROSA)26Sor*^{tm4} mice to NOD mice for five generations. A genome wide scanning including 66 microsatellite marker loci displaying allelic differences between the B6 donor genome and the NOD recipient genome with an average span of 18.3 cM between loci was carried out (ESM table 1). This established that the NOD genome was fixed in all chromosomes except for chromosome 6 (♦) carrying the targeted locus.

ESM Data to Nilsson J et al:

ESM Table 1: Microsatellite markers and loci for genotyping

NR	Namn	Chr	cM	B6	NOD		NR	Namn	Chr	cM	B6	NOD
#1	D1Mit430.1	1	10	119	127		#34	D9Mit2.1	9	17	191	203
#2	D1Mit380.1	1	36,9	116	114		#35	D9Mit123.1	9	42	254	258
#3	D1Mit132.1	1	43,1	150	167		#36	D9Mit350.1	9	61	131	133
#4	D1Mit102.1	1	73	86	96		#37	D10Mit213.1	10	11	260	266
#5	D1Mit155.1	1	112	264	228		#38	D10Mit115.1	10	38,4	91	93
#6	D2Mit293.1	2	11	213	236		#39	D10Mit14.1	10	65	199	193
#7	D2Mit100.1	2	47,5	119	123		#40	D11Mit2.1	11	2,4	105	120
#8	D2Mit411.1	2	77,6	135	147		#41	D11Mit143.1	11	32	87	85
#9	D2Mit148.1	2	105	114	117		#42	D11Mit289.1	11	55	165	171
#10	D3Mit178.1	3	13,8	200	202		#43	D11Mit48.1	11	77	151	144
#11	D3Mit51.1	3	35,2	244	258		#44	D12Mit182.1	12	2	181	197
#12	D3Mit57.1	3	55	167	157		#45	D12Mit143.1	12	35	157	162
#13	D3Mit19.1	3	87,6	232	242		#46	D12Mit133.1	12	56	119	105
#14	D4Mit18.1	4	5,2	221	210		#47	D13Mit13.1	13	35	157	151
#15	D4Mit17.1	4	31,4	137	123		#48	D13Mit74.1	13	59	122	85
#16	D4Mit203.1	4	60	99	105		#49	D13Mit78.1	13	75	242	220
#17	D4Mit42.1	4	81	88	84		#50	D14Mit98.1	14	3	154	170
#18	D5Mit123.1	5					#51	D14Mit60.1	14	15	120	95
#19	D5Mit146.1	5	1	116	120		#52	D14Mit75.1	14	54	186	198
#20	D5Mit309.1	5	44	139	147		#53	D15Mit13.1	15	6,7	149	153
#21	D5Mit95.1	5	68	150	162		#54	D15Mit67.1	15	40,9	183	181
#22	D5Mit143.1	5	86	111	113		#55	D16Mit107.1	16	3,4	224	228
#23	D6Mit138.1	6	0,7	195	215		#56	D16Mit139.1	16	43,1	157	162
#24	D6Mit284.1	6	37,5	207	198		#57	D16Mit153.1	16	56,8	154	158
#25	D6Mit198.1	6	67	97	111	♦	#58	D17Mit245.1	17	3	84	90
#26	D6Mit373.1	6	74,3	175	187		#59	D17Mit51.1	17	22,9	161	157
#27	D7Mit267.1	7	11	202	188		#60	D17Mit93.1	17	44,5	163	151
#28	D7Mit350.1	7	41	223	245		#61	D18Mit222.1	18	6	208	120
#29	D7Mit101.1	7	60	107	115		#62	D18Mit91.1	18	29	137	135
#30	D8Mit155.1	8	1	156	152		#63	D18Mit144.1	18	57	183	179
#31	D8Mit211.1	8	49	155	164		#64	D19Mit68.1	19	6	121	107
#32	D8Mit88.1	8	58	131	144		#65	D19Mit90.1	19	41	130	134
#33	D9Mit90.1	9	9	116	112		#66	D19Mit33.1	19	53	269	237

ESM Data to Nilsson J et al:

ESM Table 2: Checklist for Reporting Human Islet Preparations Used in Research

Adapted from Hart NJ, Powers AC (2018) Progress, challenges, and suggestions for using human islets to understand islet biology and human diabetes. Diabetologia https://doi.org/10.1007/s00125-018-4772-2

Islet preparation	1	2	3	4	5
MANDATORY INFORMATION					
Unique identifier	258	247	243	238	229
Donor age (years)	34	67	67	73	41
Donor sex (M/F)	M	M	M	F	M
Donor BMI (kg/m2)	26,3	32,5	26,1	22,3	24,5
Donor HbA1c or other measure of blood glucose control	5,3	5,8	5,6	5,8	5,6
Origin/source of isletsb	LUDC Malmö	LUDC Malmö	LUDC Malmö	LUDC Malmö	LUDC Malmö
Islet isolation centre	Uppsala	Uppsala	Uppsala	Uppsala	Uppsala
Donor history of diabetes? Yes/No	No	No	No	No	No
RECOMMENDED INFORMATION					
Donor cause of death					
Warm ischaemia time (h)					
Cold ischaemia time (h)					
Estimated purity (%)	78	60	82	45	55
Estimated viability (%)					
Total culture time (h)d	4	3	4	5	3
Glucose-stimulated insulin secretion or other functional measuremente	2.6	9.3	8.6	5.7	17.4
Handpicked to purity? Yes/No	Yes	Yes	Yes	Yes	Yes

ESM Data to Nilsson J et al:

ESM Table 3:

**Primary Antibodies**			
**rat anti-mouse CD31**	BD Biosciences	BD 550274	1:200
**rat anti-human CD31**	BD Biosciences	BD 561654	5µl per test
**rat anti-mouse CD45-APC/Cy7**	BioLegend	103116	1:150
**rat anti-mouse Sca-1 (Ly-6A/E)**	BioLegend	122512	1:100
**guinea pig anti-swine Insulin**	Agilent, DAKO	A0564	1:400
**rabbit anti-GFAP**	Agilent, DAKO	Z0334	1:200
**rat anti-human somatostatin**	BioRAD	8330-0009	1:200
**hamster anti-Podoplanin/gp38**	Abcam	ab11936	1:100
**rabbit anti-swine Glucagon**	EuroProxima	2263B31-1	1:200
**rabbit anti-pan laminin**	Sigma	L9393	1:200
**rabbit anti-αSMA**	Abcam	ab5694	1:200
**rabbit anti-collagen I**	Abcam	ab21286	1:200
**rabbit anti-collagen IV**	Abcam	ab19808	1:200
**rat anti-perlecan**	Merck Millipore	MAB1948P	1:400
**rabbit anti-NG2**	Merck Millipore	AB 5320	1:400
**rabbit anti-pan cytokeratin**	Santa cruz	Sc-15367	1:100
**rabbit anti-vimentin**	Abcam	ab92547	1:200
**anti-mouse CD324 (EpCAM)**	Biolegend	118210	1:200
**anti-human CD324 (EpCAM)**	Biolegend	324209	5µl per test
**rat anti-PDGFRβ (CD140b)**	Biolegend	136009	1:200
**rat anti- PDGFRα (CD140a)**	Biolegend	135901	1:200
**rat anti CD324 (E-caherin)**	Biolegend	147308	1:200
**rabbit anti-Laminin α5**	Korpos *et al.*,2013		1:200
**rabbit anti-Laminin γ1**	Korpos *et al.*,2013		1:200

ESM Data to Nilsson J et al:

ESM Fig 1

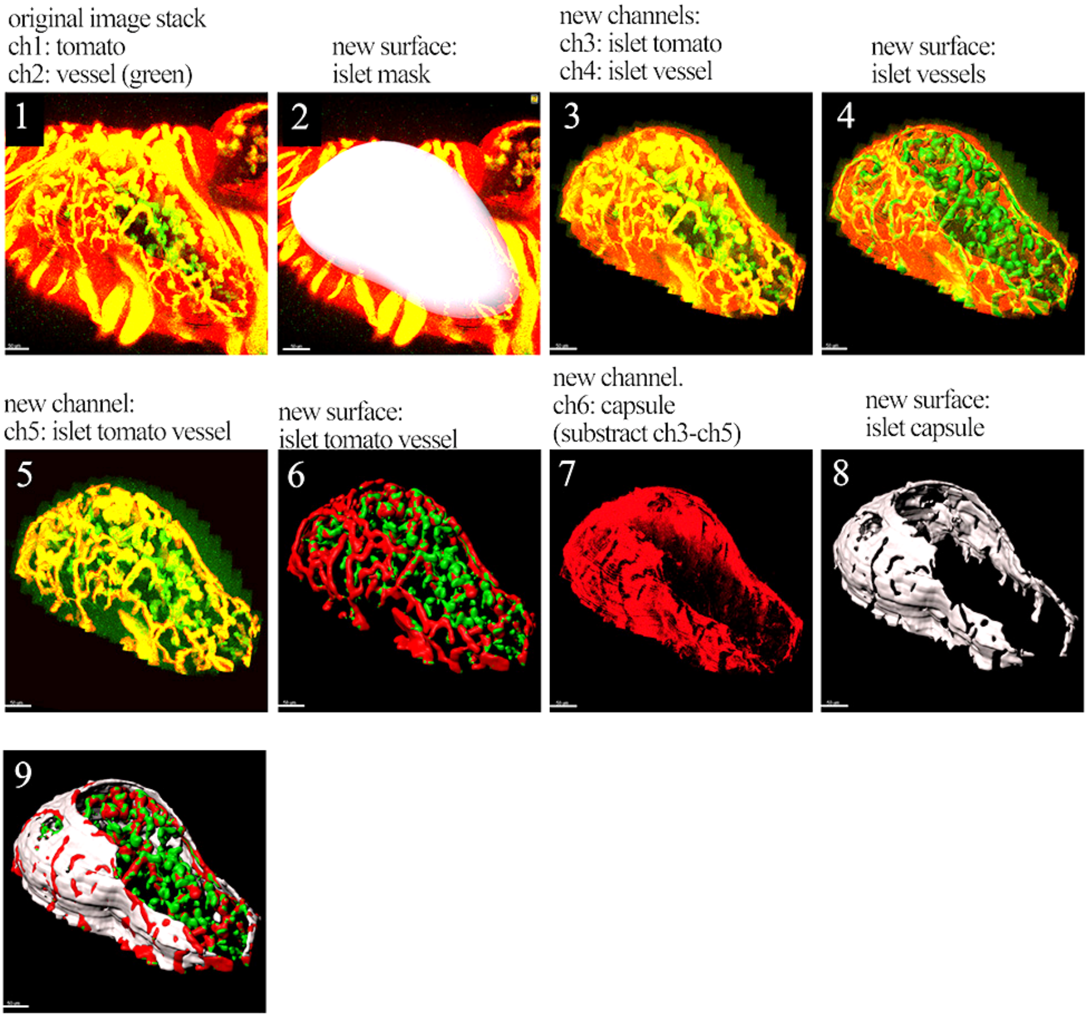

ESM Fig 1: **Image analysis using Imaris 9.1**
Cellular and vascular fractions were quantified from median filtered images within the top 75 µm. The variables reported here include *total islet volume* (based on absolute intensity of backscatter signal), *islet vessel volume in percent* (based on islet vessel volume detected by FITC/Angiosense 680 as a fraction of islet volume detected by backscatter light in the top 75µm of the image stack), *capsule in percent* (based on the tomato capsule external surface area as a fraction of the total islet surface area excluding the bottom surface area of the islet) and *ratio tomato versus total islet vascular volume* (based tomato islet vessel surface area as a fraction of the total islet vessel surface area detected by FITC/Angiosense 680). To apply measurements to just the vessels of the islets we used the Mask Channel option. The Mask Channel option and the Channel Arithmetic's option were used to subtract channels and separate the tomato channel into tomato capsule and tomato islet vessel channels for further calculations.

ESM Fig 2

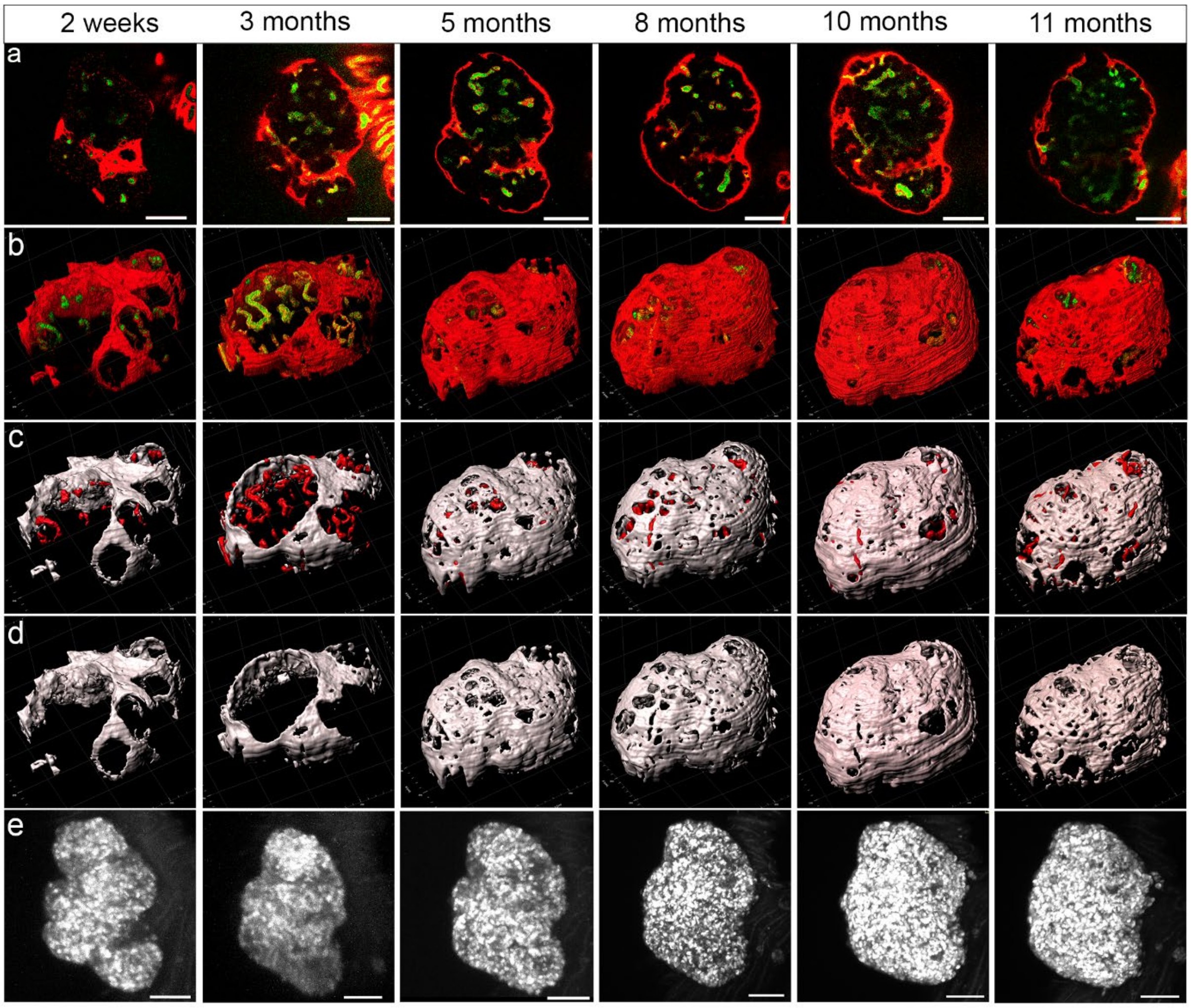

ESM Fig. 2: Transplanted islets of human origin are progressively encapsulated by mT+ cells of recipient origin. Human islets transplanted into the anterior eye chamber of NOD.*ROSA*-tomato.*Rag2*$^{-/-}$ recipient mice were imaged *in vivo* repeatedly for up to 11 months. One representative islet is shown at the time points of imaging as indicated. Optical z-sections (original recording) **(a)** or three-dimensional reconstructions of islets (**b**) with total islet vasculature (green; iv injection of fluorescent agent) recipient derived mT^{+} vasculature and mT^{+} capsule (red) at indicated time points post-transplantation. **c-d**: Image Stack segmentation (surface rendering) illustrating encapsulating mT^{+} cells in grey and mT^{+} blood vessels in red (**c**) or mT^{+} capsule alone (**d**). **e**: Confocal images displayed as maximum intensity projections (MIPs) of optical Z-stacks of backscatter light (gray). **b-d**: Image segmentation has been used on original recordings for visualization purposes (e.g. excluding signal from the iris). Scale bars, 100µm.

ESM Figure 3

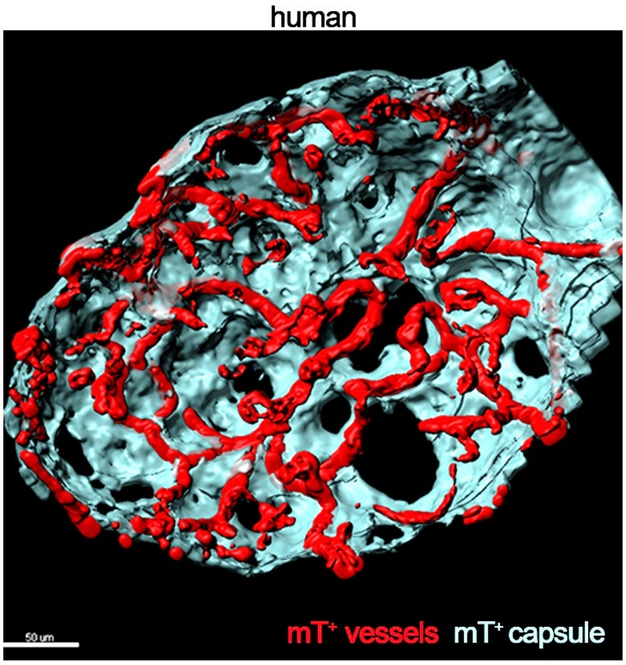

ESM Fig. 3: 3D surface rendering of total islet mT$^+$ cell signal and separation into mT$^+$ host cell derived capsule and mT$^+$ islet vascular surface. Image Stack segmentation related to Fig 2l (human islet graft-in vivo) illustrating encapsulating mT$^+$ cells in grey and mT$^+$ blood vessels in red. Ventral view.

ESM Figure 4

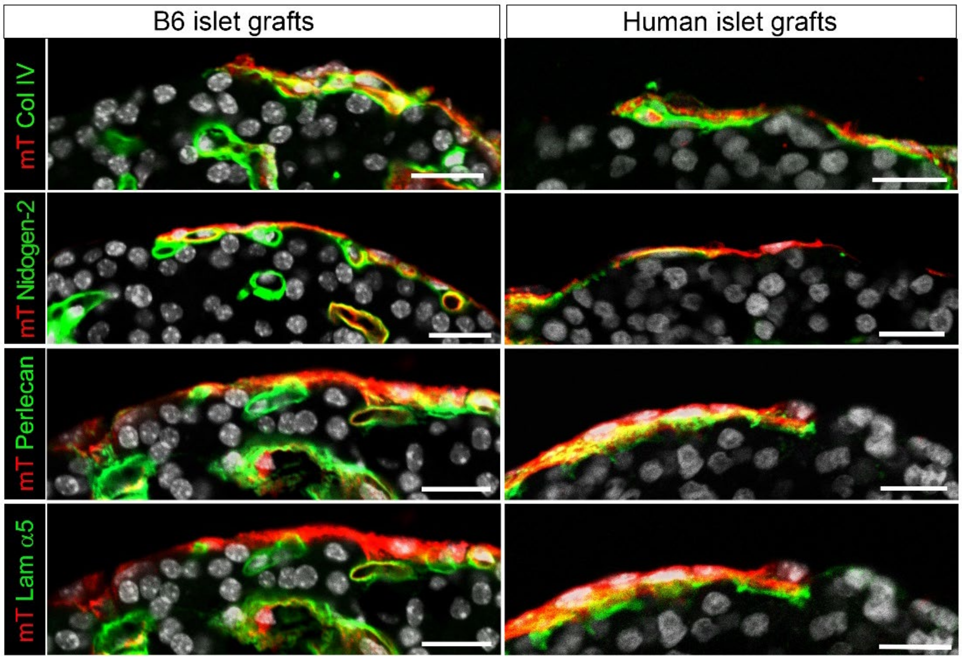

ESM Fig. 4: Encapsulating host cell-derived mT$^+$ cells correlate with the secretion of ECM proteins into a BM-like structure. Cryosections of eyes from B6.*ROSA*-tomato or NOD.*ROSA*-tomato.*Rag2*$^{-/-}$ recipient mice transplanted with either B6-albino or human islets at 3-5 months post transplantation counterstained with DAPI (grey) and collagen IV, nidogen-2, perlecan or laminin α5. Scale bars: 20µm

ESM Figure 5

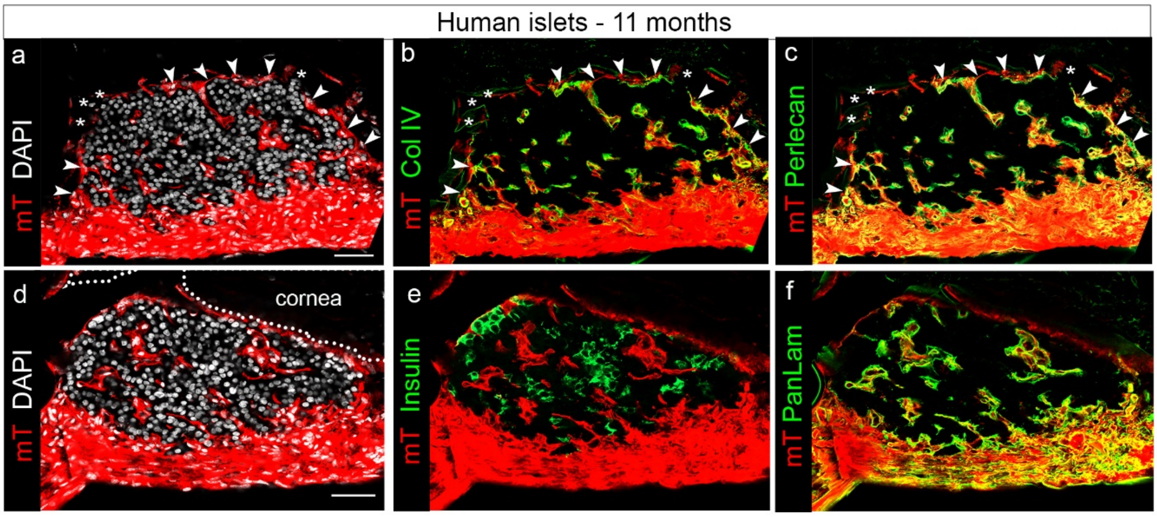

ESM Fig 5: Encapsulating host cell-derived mT$^+$ cells correlate with the secretion of ECM proteins in human islet grafts.
Cryosections of NOD.*ROSA*-tomato.*Rag2*$^{-/-}$ recipient mice bearing human islet grafts at 11 months post transplantation (related to ESM Fig 2, imaging end point) were counterstained with nuclear marker DAPI (**a, d**) in grey or collagen IV (**b**), Perlecan (**c**) Insulin (**e**) or pan Laminin (**f**) in green. The arrows indicate recipient cells (in **a**: DAPI$^+$ mT$^+$, in **b,c**: mT$^+$) ensheathing the islet and surface of islet grafts. The colocalization of mT$^+$ cells (red) and ECM (green) appears in yellow, and asterix indicates the islet surface lacking mT$^+$ cells. Scale bar: 50µm.

ESM Figure 6

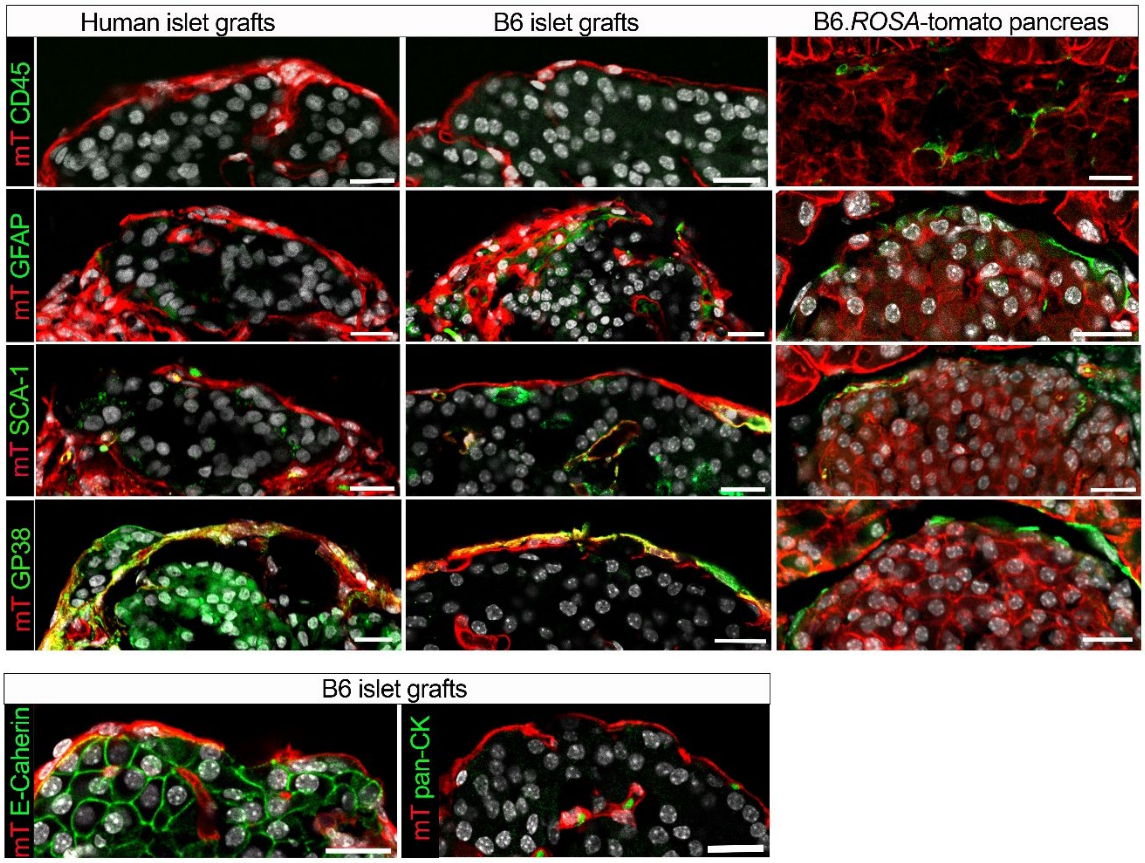

ESM Fig 6: **Recruited encapsulating mT$^+$ cells from the host are not of myeloid, Schwann cell, mesenchymic stem cells (MSC) or epithelial cell origin, but share features of fibroblast cells.** Cryosections of recipient NOD.*ROSA*-tomato.*Rag2*$^{-/-}$ eyes with human islet grafts or B6.*ROSA*-tomato eyes with B6 islet grafts 3-5 months post-transplantation or recipient B6.*ROSA*-tomato pancreas showing endogenous membrane-targeted tomato expression (mT, red) and DAPI stain (grey) counterstained (in green) with leukocyte/myeloid marker CD45, Schwann cell marker GFAP, MSC marker Sca-1, epithelial marker E-Caherin, pan-cytokeratin or the reticular fibroblast marker podoplanin/gp38. Yellow indicates co-localization of mT+ cells with green staining. Scale bars: 20 µm. Relating to Figure 3.

ESM Data to Nilsson J et al:

ESM Video 1

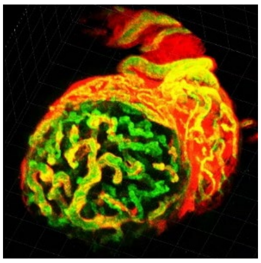

ESM Video 1: Contribution of donor and mT$^+$ recipient cells in the islet revascularisation of a syngeneic mouse islet graft 8 month post transplantation (relating to Figure 6a). Three dimensional rendering showing vessel volume in green (by i.v. injection of imaging agent) and mT$^+$ recipient cells visualised by membrane-targeted and constitutively expressed membrane targeted Tomato in red. For visualisation purpose the total mT$^+$ cell population was separated into mT$^+$ cells of the islet capsule (identified as fibroblasts) and vessel associated mT$^+$ recipient cells. Shown is a chimeric pattern of green and yellow (merge of red and green) vessel surfaces. Green vessel surface indicates donor origin and red or yellow vessel surface indicates recipient cell origin.

ESM Video 2

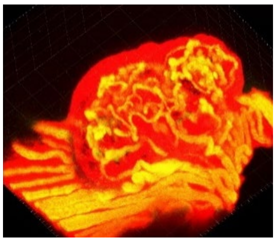

ESM Video 2: Contribution of donor and mT$^+$ recipient cells in the islet revascularisation of a human islet graft 8 months post transplantation (relating to Figure 6). Three dimensional rendering showing vasculature in green and mT$^+$ recipient cells in red. For visualisation purpose the total mT$^+$ cell population was separated into mT$^+$ cells of the islet capsule (identified as fibroblasts) and vessel associated mT$^+$ recipient cells. Red or yellow (merge of red and green) vessel surface indicates recipient cell origin.
